# Postbiotics as Next Generation Biotherapeutics Targeting the Gut–Immune–Metabolic Axis: An Integrative Review

**DOI:** 10.3390/ph19081184

**Published:** 2026-07-28

**Authors:** Asad Abbas, Ralf Weiskirchen, Muhammad Bilal, Muhammad Khurram Afzal, Abdul Malik, Suhail Akhtar, Masooma Khan, Izma Rashid, Fatima Khalid, Shazia Akram, Anza Saleem, Stanley Irobekhian Reuben Okoduwa

**Affiliations:** 1 Department of Human Nutrition, Faculty of Food Science and Nutrition, Bahauddin Zakariya University, Multan 60800, Pakistan; asadabbaskhichi@gmail.com (A.A.); shaziaakram2001@gmail.com (S.A.); 2 Institute of Molecular Pathobiochemistry, Experimental Gene Therapy and Clinical Chemistry (IFMPEGKC), RWTH University Hospital Aachen, D-52074 Aachen, Germany; 3 Faculty of Food Science and Nutrition, Bahauddin Zakariya University, Multan 60800, Pakistan; sheikhbilal4666@gmail.com; 4 Department of Pharmaceutics, College of Pharmacy, King Saud University, Riyadh 11451, Saudi Arabia; amoinuddin@ksu.edu.sa; 5 Department of Biomedical Education and Data Science Lewis Katz School of Medicine, Temple University, Philadelphia, PA 19140, USA; suhail.akhtar@temple.edu; 6 Multan College of Nutritional Sciences, Multan Medical and Dental College, Multan 60000, Pakistan; masoomakhan1112@gmail.com (M.K.); izmarashid2002@gmail.com (I.R.); anzasaleem3@gmail.com (A.S.); 7 Department of Research Innovation Management, Directorate of Research and Development, Nigerian Institute of Leather and Science Technology (NILEST), Zaria 810106, Nigeria; 8 World Nutrition Editorial Office, World Public Health Nutrition Association, London SO16 6YD, UK

**Keywords:** endocrine regulation, gut barrier integrity, immunomodulation, metabolic syndrome, microbiota–host crosstalk, oxidative stress, postbiotics, short-chain fatty acids

## Abstract

The gut–immune–metabolic axis has emerged as a central regulator of human health, with growing evidence indicating that microbiota-derived metabolites improve gut microbial ecology, enhance intestinal barrier integrity, reduce systemic inflammation, and maintain metabolic homeostasis. This review synthesizes current mechanistic and clinical evidence on the role of postbiotics in regulating intestinal barrier integrity, immune responses, oxidative stress, and metabolic–endocrine homeostasis. The literature was identified through the PubMed/MEDLINE, Scopus, and Web of Science, integrating evidence from experimental, mechanistic, animal and clinical studies on the therapeutic potential of postbiotics to modulate the gut–immune–metabolic axis. Preclinical studies suggest that postbiotics may enhance epithelial barrier function by improving tight junction integrity through multiple pathways such as PI3K/Akt signaling, stimulating mucin-2 (MUC2) production, and reducing intestinal permeability. They modulate immune responses through interactions with Toll-like receptors, nucleotide-binding oligomerization domain receptors, and G-protein-coupled receptors (GPR41/43), influencing key signaling pathways, including NF-κB and Nrf2, and altering cytokine profiles, such as IL-10, TNF-α, and IFN-γ. Similarly, preclinical investigations have demonstrated that short-chain fatty acids (SCFAs) and other microbial metabolites may improve insulin sensitivity, regulate hepatic gluconeogenesis, stimulate glucagon-like peptide 1 (GLP-1) secretion, and modulate lipid metabolism through the FXR and TGR5 signaling pathways. Emerging human studies suggest potential benefits of postbiotics in regulating gut, immune, and metabolic health; nevertheless, clinical evidence remains limited and is influenced by variability in postbiotic composition, dosage, formulation, and metabolite profiles. Therefore, standardized production approaches and well-designed large-scale randomized clinical trials are required to confirm therapeutic efficacy and establish evidence-based applications of postbiotics.

## 1. Introduction

Postbiotics have emerged as a new frontier in food, nutrition, and pharmaceutical research, representing an emerging class of fermentation-derived bioactive compounds with defined molecular mechanisms that bridge food microbiology and host metabolic regulation [1]. The human gastrointestinal tract harbors a complex and dynamic microbial ecosystem that functions as a metabolically active organ influencing immune homeostasis, endocrine signaling, and systemic metabolic regulation [2,3]. Increasing evidence suggests that the gut microbiota functions as an “endocrine-like” entity that communicates with host tissues via bioactive metabolites, structural components, and signaling molecules [4,5]. Conversely, disruption of microbial homeostasis, commonly referred to as gut dysbiosis, has been closely linked to chronic inflammatory and metabolic disorders, including obesity, type 2 diabetes mellitus, metabolic syndrome, inflammatory bowel disease (IBD), and immune-mediated conditions [6,7].

Contemporary environmental and lifestyle factors, including the widespread consumption of ultra-processed foods [8], excessive antibiotic use [9], chronic psychological stress [10], and physical inactivity [11], have been implicated in the disruption of gut microbial homeostasis. These factors contribute to increased intestinal permeability, low-grade systemic inflammation, and metabolic endotoxemia, thereby facilitating the development and progression of metabolic and inflammatory disorders [8,9,10,11]. Historically, microbiome-targeted interventions have primarily focused on probiotics, and prebiotics [12]. Despite their therapeutic potential, probiotics are associated with several limitations, including strain-specific variability, reduced viability during processing and gastrointestinal transit, storage instability, and potential safety concerns in immunocompromised individuals [13,14].

These limitations have driven increasing scientific and clinical interest in postbiotics, an emerging class of microbiome-derived bioactive preparations with well-defined functional properties. The International Scientific Association for Probiotics and Prebiotics defines postbiotics as “preparations of inanimate microorganisms and/or their components that confer a health benefit on the host, whereas prebiotics are substrates selectively utilized by host microorganisms that also confer a health benefit [15]. Moreover, postbiotics are preparations of inactivated microorganisms and/or their cellular components that provide physiological benefits to the host [16]. Importantly, purified metabolites such as isolated short-chain fatty acids (SCFAs) or exopolysaccharides alone are generally not considered postbiotics unless they are part of a defined microbial preparation derived from microbial fermentation processes [17]. Postbiotics also include functional molecules such as bacteriocins, peptidoglycans, lipoteichoic acids, microbial-derived extracellular vesicles, and cell wall fragments [18]. Unlike live probiotics, postbiotics do not require viability for efficacy. Their advantages include enhanced stability, longer shelf life, easier storage, defined molecular composition, and reduced risk of bacterial translocation or infection [19]. Importantly, postbiotics exert direct mechanistic effects on host signaling pathways, including the modulation of epithelial barrier proteins, immune cell activation, metabolic enzyme regulation, and oxidative stress responses [20,21].

Mechanistically, postbiotics influence intestinal barrier function by enhancing tight junction (TJ) proteins, such as occludin and claudin, and by promoting mucin production [22]. They regulate immune signaling via pattern recognition receptors, including TLRs and NOD-like receptors, affecting NF-κB, MAPK, and inflammasome pathways [23,24]. Additionally, microbial metabolites, such as SCFAs, activate GPR41, GPR43, and GPR109A, stimulating the release of gut hormones, including GLP-1 and PYY, thereby influencing appetite, insulin secretion, and glucose metabolism [25,26].

Emerging evidence further highlights the role of postbiotics in bile acid metabolism, lipid regulation, antioxidant defense, and endocrine signaling pathways, positioning them as promising therapeutic agents in metabolic and immune disorders [27,28,29]. Given the rapidly expanding and multidisciplinary nature of postbiotic research, an integrative review approach was adopted to combine findings from animal studies, in vitro studies, and clinical studies, thereby providing a holistic perspective on their mechanisms of action, therapeutic potential, and future applications. Additionally, the current review provides a comprehensive and mechanistic synthesis of postbiotic effects on intestinal barrier integrity, immune regulation, oxidative stress modulation, and metabolic–endocrine homeostasis. We further evaluate clinical evidence supporting their therapeutic potential and discuss translational challenges, including standardization, regulatory frameworks, and future research directions.

## 2. Materials and Methods

### 2.1. Search Strategy

A literature search was conducted across PubMed/MEDLINE, Scopus, and Web of Science. The search was performed independently by two reviewers, and any discrepancies in retrieved records were resolved through cross verification of raw database exports. The search covered publications from January 2014 to December 2025 to gather current research on postbiotics and microbiome-targeted interventions. A combination of Medical Subject Headings (MeSH) and free-text terms was used, along with Boolean operators (AND, OR) and keywords such as “postbiotics”, “inactivated probiotics”, “heat-killed probiotics”, “metabiotics”, “paraprobiotics”, “short-chain fatty acids”, “microbial metabolites”, “gut barrier”, “intestinal permeability”, “immune modulation”, “NF-κB”, “Nrf2”, “oxidative stress”, “glucose metabolism”, “lipid metabolism”, “metabolic syndrome”, “type 2 diabetes”, “obesity”, and “inflammatory bowel disease”. Search terms were organized into four conceptual blocks linked by Boolean operators to improve precision and reproducibility. A sample PubMed search string was structured as follows: (i) postbiotic identity by “postbiotics” OR “heat-killed probiotics” OR “inactivated probiotics” OR “tyndallized probiotics” OR “paraprobiotics” OR “metabiotics” OR “inactivated microorganisms” OR “cell-free supernatant”. (ii) Molecular components searched by “short-chain fatty acids” OR “SCFA” OR “butyrate” OR “propionate” OR “microbial metabolites” OR “peptidoglycan” OR “lipoteichoic acid” OR “exopolysaccharides”. (iii) Mechanistic outcomes by “gut barrier” OR “intestinal permeability” OR “tight junction” OR “ZO-1” OR “occludin” OR “immune modulation” OR “cytokines” OR “NF-κB” OR “Nrf2” OR “oxidative stress” OR “superoxide dismutase” OR “malondialdehyde”. (iv) Clinical and metabolic outcomes by “metabolic syndrome” OR “diabetes” OR “obesity” OR “lipid metabolism” OR “glucose metabolism “OR “insulin resistance” OR “lipid profile” OR “inflammatory bowel disease”.

### 2.2. Eligibility Criteria

#### 2.2.1. Inclusion Criteria

Original research articles, including human clinical trials, animal studies, and mechanistic in vitro studies, were included if they evaluated defined postbiotic preparations, including inactivated microbial cells, cell wall components, secreted microbial metabolites, and cell-free supernatants, in accordance with the ISAPP 2021 consensus definition [15]. The following aspects were evaluated in each included studies: (i) postbiotic characterization including inactivation method, dose, and route; (ii) adequacy of control conditions; (iii) validation of outcome measurement assays; (iv) appropriateness of statistical methods; and (v) indicators of reproducibility, including replicates in in vitro studies, animal numbers in preclinical studies, power estimates, registration status, and sample size justification in clinical studies.

#### 2.2.2. Exclusion Criteria

Studies on live probiotics without postbiotic characterization, used incompletely characterized fermentation products, undefined lysates, and preparations lacking documented microbial origin were excluded. Moreover, the review articles, commentaries, editorials, or conference abstracts that did not report measurable biological or clinical outcomes or lacked a clear description of postbiotic preparation or composition were also excluded.

**Figure 1:**
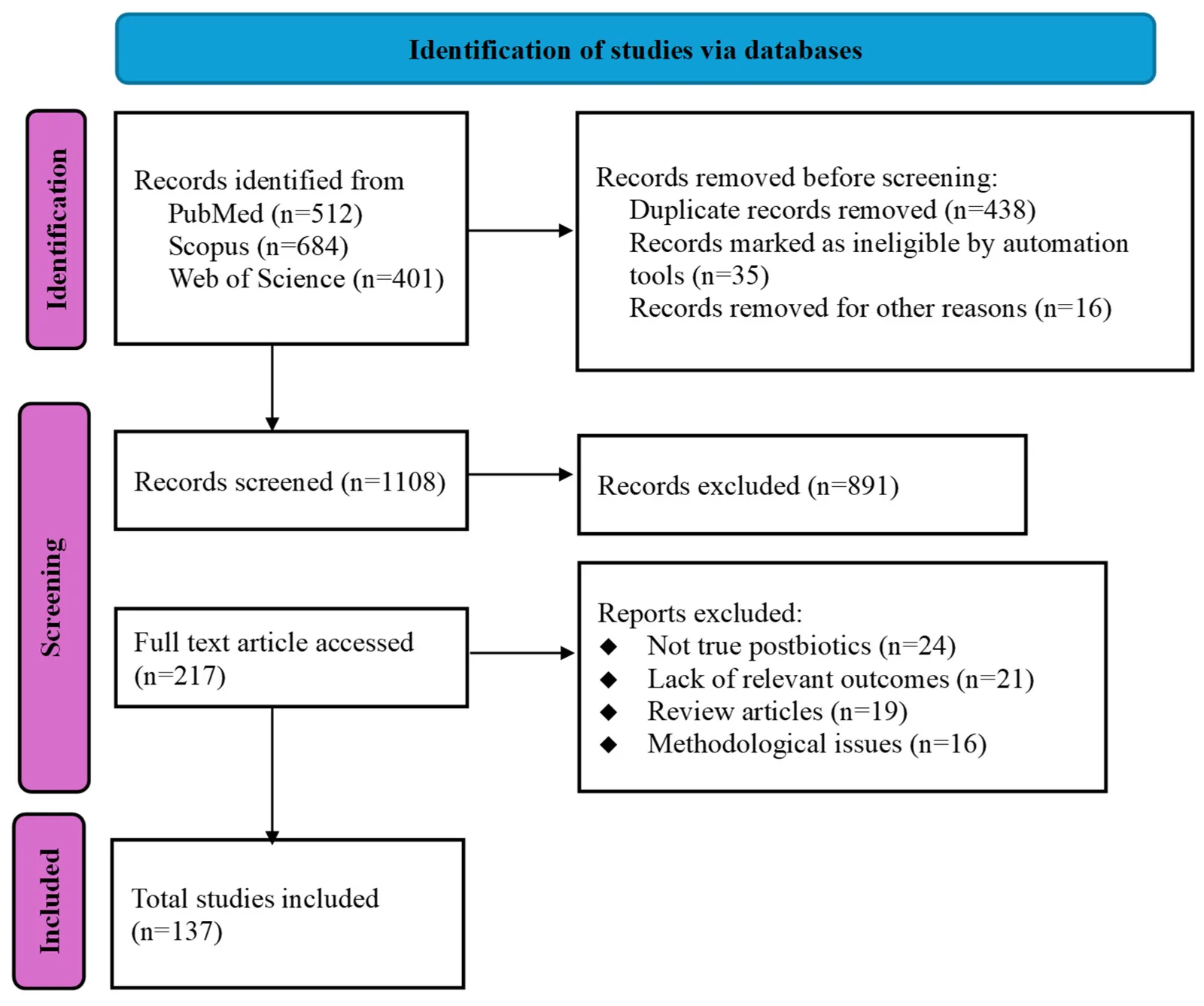
Flow diagram illustrating the study selection process used in this systematic review. Database searches in PubMed/MEDLINE, Scopus, and Web of Science identified 1597 records. After removing duplicates and screening titles and abstracts, 217 articles were assessed as full-text articles, with 134 studies ultimately included in the qualitative synthesis. These studies evaluated the mechanistic and clinical effects of postbiotics on intestinal barrier integrity, immune modulation, oxidative stress regulation, and metabolic–endocrine disorders.

Database searching revealed 1597 records from databases such as PubMed (*n* = 512), Scopus (*n* = 684), and Web of Science (*n* = 401). After eliminating duplicate records, the screened records totaled 1159. Title and abstract screening resulted in the elimination of 942 records due to irrelevance to the study objectives. As a result, 217 full-text articles were evaluated for eligibility. Among these, 80 articles were eliminated for reasons such as the use of only live probiotics (*n* = 24), absence of relevant clinical or mechanistic outcomes (*n* = 21), review articles (*n* = 19), and inadequate methodological descriptions (*n* = 16). Finally, 137 studies were included in the qualitative synthesis. The study selection process is summarized in the schematic flow diagram shown in Figure 1.

## 3. Composition and Bioactive Components of Postbiotics

Postbiotics are diverse in terms of biologically active compounds obtained from cell-free supernatants (CFS) and heat-killed/inactivated bacterial cells (HKB), which play a synergistic role in their functional health effects [30]. Major peptide components in postbiotic supernatants include *bacteriocins*, quorum-sensing peptides, proteases such as lactocepin [31,32], cell wall hydrolases (p40 and p75), and extracellular proteins [33]. Additionally, they contain bioactive lipids (e.g., SCFAs like butyrate and conjugated linoleic acid) [34], exopolysaccharides [35], small organic molecules (e.g., lactic acid, D-amino acids, and indole derivatives), vitamins, cofactors, and inorganic compounds like polyphosphates [36]. Cell-derived postbiotics contain structural and intracellular elements such as S-layer proteins, lipoteichoic acid, peptidoglycan-derived muropeptides [20], polysaccharides like galactose-rich capsular polysaccharides and teichoic acids [37], as well as intracellular proteins and fermentation-derived metabolites. The combination of these compounds creates a complex mixture that contributes to the immunomodulatory, metabolic, and therapeutic potential of postbiotics.

## 4. Mechanistic Foundations of Postbiotics

Postbiotics exert pleiotropic biological effects by directly interacting with host epithelial, immune, and metabolic signaling pathways [20]. They act primarily through defined microbial structural components and bioactive metabolites. These molecules interact with host receptors, intracellular signaling cascades, and epigenetic regulatory mechanisms to modulate physiological responses [38]. Rather than functioning through isolated mechanisms, postbiotics appear to act within an interconnected pathophysiological framework in which intestinal barrier dysfunction, microbial product translocation, immune activation, oxidative stress, and metabolic dysregulation reinforce one another [39]. These domains are discussed separately for clarity, but they should be interpreted as overlapping components of a shared gut–immune–metabolic axis rather than independent pathways. Current evidence suggests that several postbiotic preparations may influence metabolic regulation through multiple signaling pathways [40,41]. However, human clinical studies provide the most reliable evidence for barrier and immune modulation outcomes, whereas the majority of mechanistic explanations originate from in vitro and animal studies. While consistent biological trends are observed, the evidence base remains heterogeneous and several proposed mechanisms remain partially speculative due to limited clinical validation.

### 4.1. Intestinal Barrier Integrity

The intestinal epithelial barrier is a crucial interface between the host and the luminal microbiota. It is composed of interconnected epithelial cells held together by tight junction (TJ) proteins, including occludin, claudins, zonula occludens-1 (ZO-1), adherens junctions, and a protective mucus layer primarily composed of mucin-2 (MUC2) [42]. When this barrier is disrupted, it allows the translocation of microbial products like lipopolysaccharide (LPS) to pass into the circulation, contributing to metabolic endotoxemia and systemic inflammation [43].

Postbiotics play a role in enhancing epithelial barrier integrity by activating intracellular signaling pathways that regulate the expression of TJ proteins [44]. Studies have shown that postbiotic preparations activate the PI3K/Akt pathway, leading to increased expression of occludin and claudin proteins, ultimately reducing intestinal permeability. This signaling cascade helps strengthen epithelial cohesion and decrease paracellular leakage [45,46]. These mechanistic findings are primarily derived from preclinical models, and clinical confirmation remain limited. Additionally, other groups have reported that microbial-derived metabolites can improve TJ stability and reduce permeability during inflammatory conditions [47], although the magnitude and consistency of this effect vary depending of postbiotic preparation. Strengthening TJ architecture is especially important in cases of obesity and metabolic syndrome, where gut permeability is elevated [48]. They also stimulate mucin production and enhance MUC2 gene expression, leading to thicker mucus layers and improved mucosal protection [49]. This process is partly mediated through the modulation of NF-κB signaling, which plays a role in both maintaining the barrier and regulating inflammatory responses [20,38].

Importantly, clinical evidence supports gut barrier related benefits such modulation of epithelial barrier integrity, immune responses, and inflammatory signaling pathways [50,51]. For instance, a clinical study demonstrated that supplementation with ReFerm^®^-containing an inactivated microbial cell matrix and metabolites, significantly improved intestinal permeability in patients with alcoholic fatty liver disease and improved markers associated with fibrosis progression [50]. In patients with diarrhea-predominant irritable bowel syndrome (IBS-D), both live and heat-treated *Bifidobacterium longum (B. longum)* CECT 7347 reduced symptom severity and improved stool consistency [51], indicating clinical evidence of barrier stabilization to improve inflammatory markers. Overall, these evidences strongly support a barrier-protective trend for strengthening both the structural and functional aspects of the intestinal barrier, reducing endotoxin translocation and chronic inflammatory burden.

### 4.2. Immune Modulation

The gut-associated lymphoid tissue (GALT) is tightly regulated by microbial-derived signals. Postbiotics interact directly with pattern recognition receptors (PRRs), including Toll-like receptors (TLRs) and nucleotide-binding oligomerization domain (NOD) receptors, influencing innate and adaptive immune responses. Preclinical studies documented that postbiotic components such as peptidoglycans, lipoteichoic acids, and bacteriocins engage TLRs and NOD-like receptors, initiating downstream signaling cascades involving NF-κB and MAPKs [21,23]. This activation modulates cytokine production and immune cell differentiation. Activation of TLRs promotes balanced immune responses rather than uncontrolled inflammation. Furthermore, TLR signaling orchestrates innate immune activation while maintaining tolerance [52]. Meanwhile, SCFA-sensitive G-protein-coupled receptors (FFA2 and FFA3) modulate immune signaling and enhance regulatory T cell activity [53,54]. However, immune responses are context dependent and both pro- and post-inflammatory signaling may be observed depending on dose, strain, and preparation type [20].

Postbiotics may influence cytokine balance by suppressing pro-inflammatory mediators (TNF-α, IL-6, IL-1β) while promoting anti-inflammatory cytokines such as IL-10. Moreover, modulation of cytokine ratios favors immune tolerance [24,55]. This cytokine modulation is relatively well supported across both animal and limited human studies, representing one of the more consistent findings in the literature. In contrast, modulation of NF-κB (pro-inflammatory) and Nrf2 (antioxidant) pathways, resulting in reduced inflammatory gene expression in high-fat diet-induced animal models [56,57]. The NF-κB pathway comprises intracellular pathways that control inflammatory responses through innate and adaptive mechanisms [58]. The NF-κB family of transcription factors consists of five mammalian members: RelA (p65), RelB, c-Rel, NF-κB1 (p50/p105) and NF-κB2 (p52/p100) [59,60]. Postbiotics have profound immunomodulatory effects because they influence major signaling pathways of inflammation through the NF-κB pathway [45], but their effects can vary based on the bacterial strain, source, and whether the preparation is CFS or HKB [20]. Postbiotics may tend to down-regulate inflammation by inhibiting the phosphorylation of IκB, preventing its degradation, and consequently blocking the nuclear translocation of NF-κB and the subsequent expression of pro-inflammatory genes [56,61]. Simultaneously, postbiotics also regulate the MAPK signaling pathway, resulting in reduced inflammatory responses by decreasing the phosphorylation of kinases including p38 [62]. They also exhibit immunostimulatory properties, stimulating the NF-κB and MAPK pathways to enhance host defense functions, such as the production of heat-shock proteins and antimicrobial peptides like β-defensin, and improving intestinal barrier integrity as documented in preclinical study [63].

**Figure 2:**
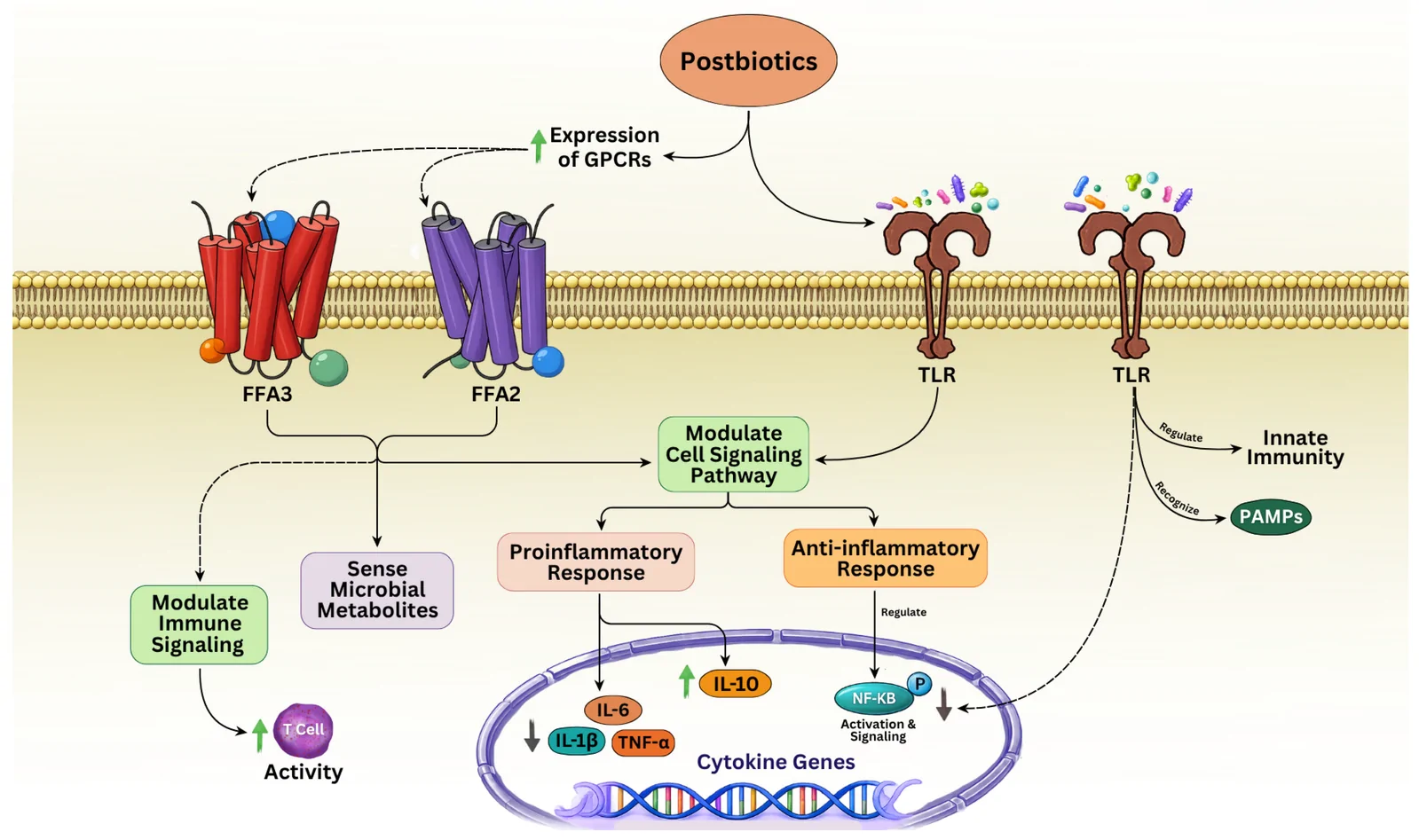
Mechanistic overview of postbiotic interactions with immune and cellular signaling pathways in the gut. Postbiotic components such as peptidoglycans, lipoteichoic acids, bacteriocins, and short-chain fatty acids interact with pattern recognition receptors, including Toll-like receptors (TLRs) and nucleotide-binding oligomerization domain (NOD) receptors. These interactions activate intracellular signaling cascades such as the NF-κB and MAPK pathways and modulate cytokine production, leading to balanced immune responses characterized by the suppression of pro-inflammatory mediators and the promotion of anti-inflammatory cytokines [21,23,52,53,54,55,56,63,64].

Similarly, heat-treated *Lactiplantibacillus plantarum* nF1 significantly increased IL-12 levels and enhanced natural killer (NK) cell activity, demonstrating improved innate immune responsiveness [64]. The increased expression of CD86 and HLA-DR on dendritic cells following postbiotic supplementation indicates enhanced antigen-presenting capacity and antiviral immune defense [65,66]. Postbiotics also regulate adaptive immune polarization, with the ability to modulate Th1/Th2 and Treg/Th17 balance, suggesting relevance in autoimmune and inflammatory disorders. Thus, postbiotics act as immunological modulators that fine-tune inflammatory responses without provoking excessive immune activation [67]. However, evidence for adaptive immune remodeling, such as long-term Th1/Th2 balance shifts, remains preliminary and should be interpreted as a potential rather than an established effect. Overall, immune modulation represents a moderately strong evidence domain, with stronger support for cytokine regulation than for long term adaptive immune reprogramming. The mechanistic role of postbiotics in modulating immune and cellular signaling pathways is illustrated in Figure 2.

### 4.3. Oxidative Stress Regulation

Oxidative stress, characterized by excessive reactive oxygen species (ROS) production, plays a central role in metabolic syndrome, IBD, and insulin resistance [68]. Postbiotics exert antioxidant effects through both direct radical scavenging and activation of endogenous antioxidant systems [69]. Postbiotic supplementation increases antioxidant enzyme activity, including superoxide dismutase (SOD), catalase (CAT), and glutathione peroxidase (GPx), while reducing malondialdehyde (MDA), a marker of lipid peroxidation [28,70]. Moreover, mixtures of postbiotics increased serum SCFAs and enhanced antioxidant defenses in aging mice, improving oxidative stress markers across multiple organs [71]. These findings suggest that postbiotics not only scavenge ROS but also restore mitochondrial bioenergetics and antioxidant homeostasis. Postbiotics significantly up-regulated *Nrf2*-related genes while suppressing NF-κB-mediated inflammatory signaling. This dual regulation highlights the capacity of postbiotics to restore redox balance and simultaneously reduce inflammation [56]. However, the integrated dual regulation of Nrf2/NF-κB is primarily mechanistic and not yet fully validated in clinical studies. Additionally, a *Lacticaseibacillus casei* CRL-431 postbiotic reduced hepatic mitochondrial oxidative stress in aflatoxin-induced models, decreasing hydrogen peroxide production and improving mitochondrial respiration efficiency [72]. The types of postbiotics, targeted effects, mechanistic pathways, evidence strength in treating metabolic disorders are shown in Table 1.

### 4.4. Microbial and Metabolic Signaling

Postbiotics exert systemic metabolic effects through microbial metabolite signaling, particularly via SCFAs, bile acid modulation, and epigenetic regulation.

#### 4.4.1. Short-Chain Fatty Acid–G-Protein-Coupled Receptor Axis

SCFAs, such as acetate, propionate, and butyrate, are key microbial metabolites that are postbiotic components. They are produced through the fermentation of dietary fibers by the gut microbiota in the colon and act as bioactive mediators linking microbial activity with host metabolic and immune regulation [83]. They have regulatory effects on host metabolism by interacting with specific G-protein-coupled receptors (GPCRs), including GPR41, GPR43, and GPR109A, expressed on intestinal epithelial and enteroendocrine cells [84]. Activation of these receptors stimulates the secretion of gut hormones such as GLP-1 and PYY), which play central roles in regulating glucose homeostasis, insulin secretion, and appetite control [31,32]. These receptor-mediated effects are well established in experimental models but only partially confirmed in human metabolic studies. Through these mechanisms, SCFAs contribute to enhanced insulin sensitivity, increased satiety, delayed gastric emptying, and reduced energy intake [85].

In addition to receptor-mediated signaling, SCFAs, especially butyrate, also have epigenetic effects by inhibiting histone deacetylases (HDACs). This inhibition promotes histone acetylation and modulates the transcription of genes involved in metabolic regulation, inflammation, and lipid metabolism [86]. However, the extent of clinically meaningful epigenetic remodeling in humans remains speculative due to limited translational evidence. Moreover, butyrate-mediated HDAC inhibition influences pathways associated with glucose utilization, fatty acid oxidation, and adipogenesis, thereby contributing to improved metabolic homeostasis [87]. Together, these receptor-dependent and epigenetic mechanisms highlight the multifaceted role of SCFAs in linking gut microbial activity with host metabolic and endocrine regulation.

#### 4.4.2. Hepatic and Lipid Regulation

SCFAs also influence host lipid metabolism by reducing lipogenic gene expression and promoting fatty acid oxidation [88]. In addition, postbiotic-mediated modulation of bile acid signaling pathways plays a crucial role in metabolic regulation [89]. Postbiotics and their derived metabolites can regulate bile acid receptors such as the farnesoid X receptor (FXR) and the G-protein-coupled bile acid receptor TGR5, thereby affecting hepatic gluconeogenesis, lipid metabolism, and energy homeostasis [27]. Through these mechanisms, postbiotics contribute to maintaining metabolic balance and preventing metabolic dysfunction.

**Figure 3:**
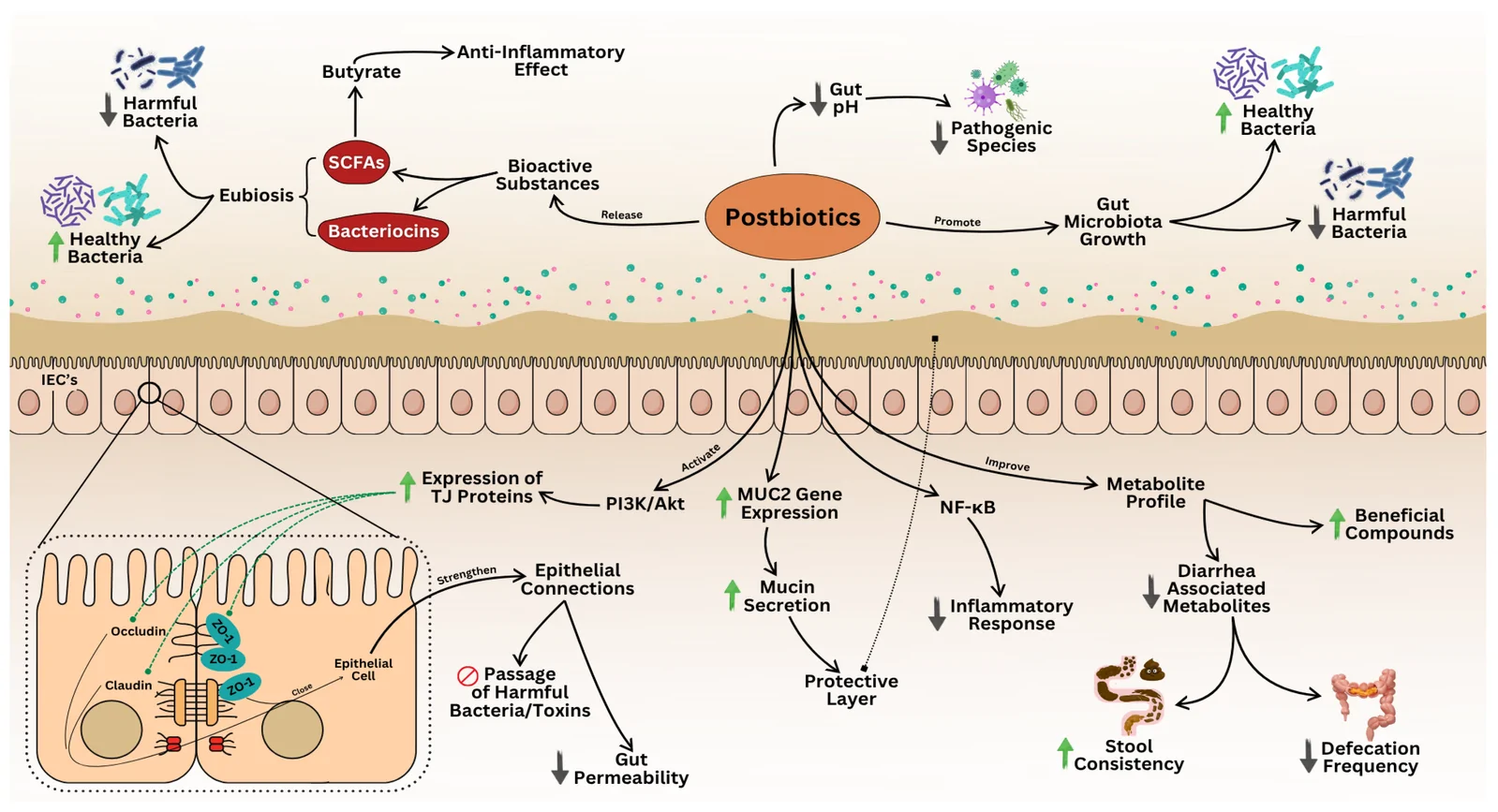
Integrated schematic representation of the molecular mechanisms by which postbiotics influence the gut–immune–metabolic axis. Postbiotic preparations, including inactivated microbial cells, short-chain fatty acids, exopolysaccharides, and microbial cell wall components, enhance intestinal barrier integrity by regulating tight junction proteins and mucin production. They modulate immune signaling through TLR/NOD-mediated activation of NF-κB and Nrf2 signaling pathways, regulate oxidative stress responses through antioxidant signaling, and influence metabolic pathways via G-protein-coupled receptors (GPR41/43), bile acid receptors (FXR and TGR5), and histone deacetylase inhibition. These combined mechanisms contribute to improved metabolic and immune homeostasis [20,31,32,56,84,85,89,90].

Postbiotic preparations, which include inactivated microbial cells, SCFAs, exopolysaccharides, and microbial cell wall components, enhance intestinal barrier integrity, modulate immune signaling pathways (such as TLR/NOD-mediated activation of NF-κB and Nrf2 pathways), regulate oxidative stress responses, and influence metabolic signaling pathways like GPR41/43 activation, FXR/TGR5 signaling, and HDAC inhibition, together contributing to improved metabolic and immune homeostasis [20,56,90]. Therefore, while postbiotics demonstrate broad biological activity, the majority of mechanistic pathways require further well-designed human clinical studies. The integrated molecular pathways through which different categories of postbiotic preparations influence intestinal barrier function, immune signaling pathways, oxidative stress regulation, and metabolic signaling networks are summarized in Figure 3.

## 5. Evidence of Postbiotics in Metabolic and Immune Disorders in Clinical and Animal Models

Emerging clinical evidence increasingly highlights the translational relevance of postbiotics in a wide range of metabolic and immune-mediated disorders [50,51]. Postbiotics represent a promising strategy for modulating host–microbiome interactions without the potential safety concerns associated with administering live microorganisms [91]. In contrast to conventional probiotics, postbiotics provide standardized bioactive preparations with improved safety profiles, enhanced stability during storage and processing, and greater mechanistic specificity [92]. These characteristics facilitate more precise therapeutic applications and support their integration into functional foods, nutraceutical formulations, and clinical interventions targeting microbiome-associated diseases [93]. Recent clinical and translational studies suggest that postbiotics may exert beneficial effects across several physiological systems, including the gastrointestinal, metabolic, immune, and hepatic axes [62,65]. Accumulating evidence indicates potential therapeutic benefits in conditions such as irritable bowel syndrome (IBS), inflammatory bowel disease, obesity, metabolic syndrome, and non-alcoholic fatty liver disease [51,57,62]. These effects are largely attributed to the capacity of postbiotic compounds, such as microbial cell wall components and exopolysaccharides, to regulate signaling pathways involved in immune activation, oxidative stress, and metabolic homeostasis [20,63].

### 5.1. Endocrine Regulation

Postbiotics modulate endocrine and metabolic functions through microbiome-mediated signaling pathways. Supplementation with a *Bifidobacterium breve*-derived postbiotic extract was shown to significantly reduce circulating inflammatory markers, including C-reactive protein (CRP) and interleukin-6 (IL-6), while simultaneously increasing levels of dehydroepiandrosterone (DHEA) and estradiol in women over 40 years of age, suggesting both anti-inflammatory and endocrine-regulatory effects [94]. These findings indicate that postbiotic compounds may influence hormonal balance by reducing systemic inflammation and modulating endocrine signaling pathways associated with aging and metabolic health. Similarly, postbiotic preparations containing γ-aminobutyric acid (GABA) or derived from GABA-producing microbial fermentation processes have demonstrated beneficial effects on reproductive endocrine function. In experimental models of stress-induced ovarian dysfunction, postbiotic preparations enriched with GABA improved ovarian hormone profiles and reduced oxidative stress markers, suggesting protective effects on ovarian physiology and endocrine homeostasis in laying hens [95]. The antioxidant and anti-inflammatory properties of these microbial metabolites may contribute to the restoration of hormonal balance by mitigating oxidative damage and regulating stress-related signaling pathways. Collectively, these findings position postbiotics as important regulators of the gut–metabolic–endocrine axis through a combination of receptor-mediated, epigenetic, and hormonal mechanisms. By influencing gut-derived signaling molecules and systemic inflammatory mediators, postbiotics may exert downstream effects on metabolic regulation, endocrine function, and immune homeostasis [96].

**Figure 4:**
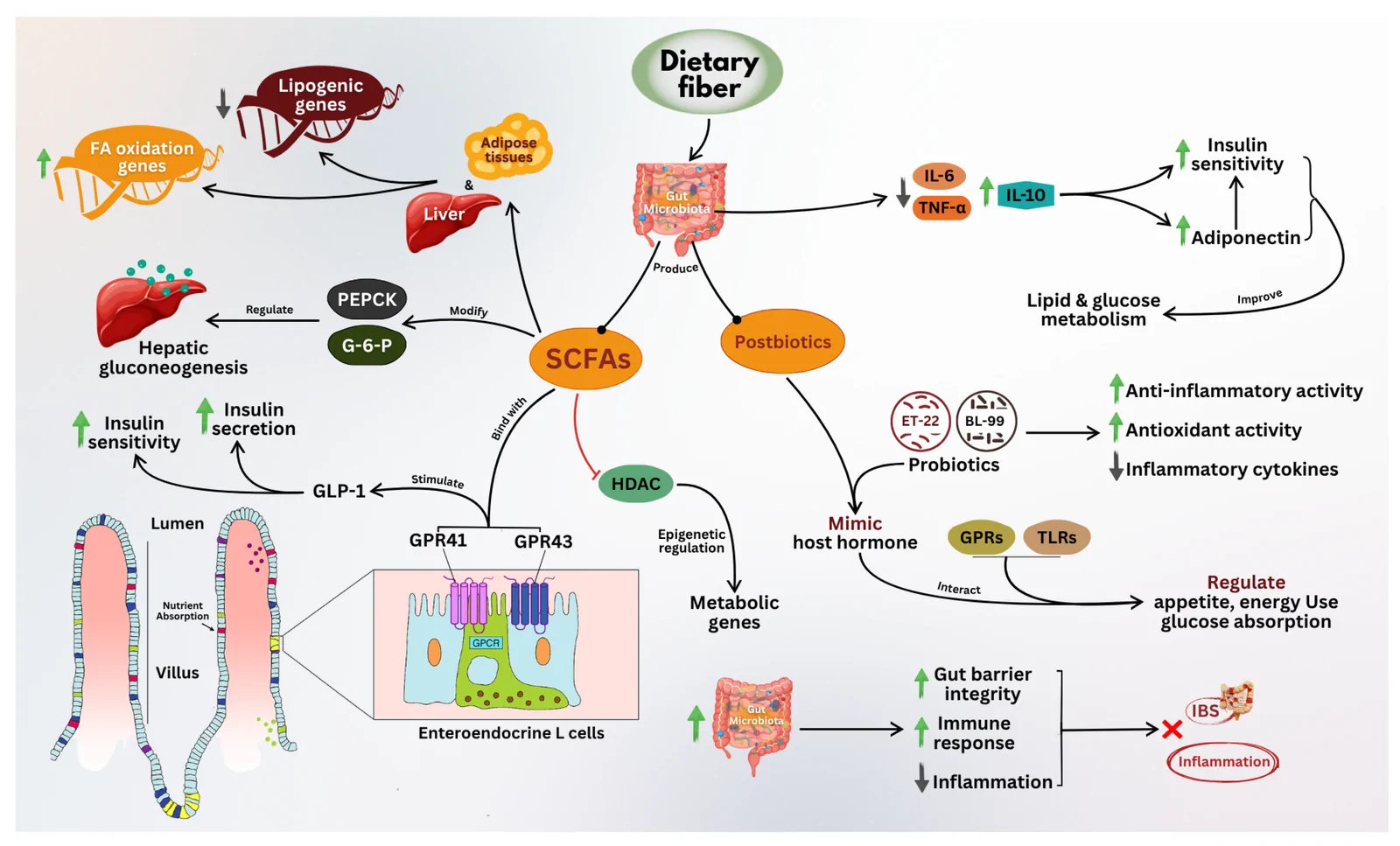
Proposed mechanisms through which postbiotics modulate endocrine functions via gut-derived signaling pathways. Dietary fiber fermentation by gut microbiota produces postbiotic metabolites, particularly short-chain fatty acids (SCFAs) such as acetate, propionate, and butyrate. These metabolites interact with intestinal receptors including G-protein-coupled receptors (GPR41/FFAR3, GPR43/FFAR2, and GPR109A) on intestinal epithelial and enteroendocrine cells. Activation of these receptors stimulates the secretion of metabolic hormones such as glucagon-like peptide-1 (GLP-1) and peptide YY (PYY), enhancing insulin secretion, improving insulin sensitivity, and regulating appetite and energy balance through gut–brain axis signaling. SCFAs also act as histone deacetylase (HDAC) inhibitors, promoting epigenetic regulation of metabolic genes involved in lipid oxidation, gluconeogenesis, and adipose tissue metabolism in the liver and peripheral tissues. In addition, postbiotic compounds interact with pattern-recognition receptors such as Toll-like receptors (TLRs) and other G-protein-coupled receptors to modulate immune signaling pathways, reducing pro-inflammatory cytokines (e.g., TNF-α and IL-6) while enhancing anti-inflammatory mediators such as IL-10. These processes contribute to improved gut barrier integrity, antioxidant defenses, and metabolic homeostasis. Through coordinated regulation of inflammatory responses, glucose and lipid metabolism, and hormone secretion, postbiotics support systemic endocrine balance and may influence reproductive endocrine pathways via the gut–endocrine axis [59,90,91,92,93,94,95,96,97].

In addition to endocrine modulation, emerging clinical evidence also highlights the potential of postbiotics in improving glycemic control and metabolic parameters. For instance, significant reductions in glycated hemoglobin (HbA1c) and fasting plasma glucose levels in prediabetic individuals following postbiotic intervention have been accompanied by an increase in butyrate-producing bacterial populations [79]. These findings suggest that postbiotic supplementation may promote beneficial shifts in gut microbial metabolism while enhancing SCFA production, which is known to improve insulin sensitivity and glucose regulation. Furthermore, Bacteroides-derived postbiotic compounds have demonstrated promising metabolic effects in obesity models. Combined postbiotic preparations have significantly reduced the expression of genes involved in hepatic lipogenesis while improving lipid and glucose metabolism [97]. These effects were associated with enhanced fatty acid oxidation and improved systemic metabolic parameters, highlighting the potential of postbiotics to modulate key pathways involved in energy balance and metabolic disease progression. Additionally, dietary fiber is fermented by the gut microbiota to produce SCFAs and other microbial metabolites, which play important roles in host metabolism. SCFAs enhance insulin sensitivity, stimulate GLP-1 secretion, and regulate glucose and lipid metabolism through receptors such as GPR41 and GPR43 [56,90]. They also influence gene expression via epigenetic mechanisms, including HDAC inhibition, leading to improved metabolic health as mentioned in Figure 4. These findings highlight the growing therapeutic potential of postbiotics as modulators of metabolic, endocrine, and inflammatory pathways, supporting their development as targeted microbiome-based interventions for metabolic disorders.

### 5.2. Irritable Bowel Syndrome

IBS, particularly diarrhea-predominant IBS (IBS-D), is characterized by visceral hypersensitivity, altered gut motility, and increased intestinal permeability. Both live and heat-treated *B. longum* CECT 7347 significantly reduced symptom severity scores in IBS-D patients. Improvements were observed in stool frequency, abdominal pain, and overall quality of life. The comparable efficacy of the heat-treated formulation suggests that microbial structural components and metabolites, rather than bacterial viability, mediate therapeutic effects [51]. Mechanistically, these clinical improvements likely arise from TJ stabilization, reduced mucosal inflammation, and modulation of gut–brain signaling pathways. The findings support the potential of postbiotics as stable alternatives to live probiotics in the treatment of functional gastrointestinal disorders.

Several clinical studies have reported complementary mechanisms by which gut health and host immunity are regulated by postbiotics [98,99,100]. Compare et al. documented the effects of postbiotics derived from *Lactobacillus casei* DG in patients with the post-infectious IBS-D (PI-IBS-D) subtype [98]. The mRNA expression of pro-inflammatory cytokines (IL-1α, IL-6 and IL-8) was significantly elevated in PI-IBS-D patients at baseline, whereas the anti-inflammatory cytokine (IL-10) expression level was significantly reduced. TLR-4 protein expression was also significantly elevated in PI-IBS-D patients at baseline. The most important mechanistic discovery was the reduction in the levels of IL-1α and IL-8, and the increase in IL-10, as well as the reduction in IL-6 levels, which were observed to a greater extent when the postbiotic was used compared with the live probiotic, indicating that microbial metabolites, rather than live bacteria alone, might be the key mediators of immunomodulatory activity. Both preparations also demonstrated reduction in the expression of TLR-4, further supporting suppression of the innate immune signaling pathway at the mucosal surface as a key mechanism in the anti-inflammatory activities of postbiotics.

In the study by Bednarska et al. [99], fermented oat gruel (ReFerm^®^) produced with *L. plantarum* 299v was studied in IBS patients with moderate-to-severe IBS-D and mixed IBS subtypes. The study demonstrated that ReFerm^®^ significantly decreased paracellular permeability and increased the transepithelial resistance (TER) in colonic biopsies from IBS patients, and that these changes were also observed in a Caco-2 cell model where both paracellular and transcellular permeability were reduced. The absence of significant differences in the effects of the heat-inactivated and live *L. plantarum* 299v on the intestinal barrier also indicates that the barrier-protective properties are exerted by the metabolites resulting from the fermentation and not by the live bacteria. Another study documented the therapeutic effects of a probiotic–postbiotic mixture in children with IBS. Postbiotic supplementation, combined with standard treatment, resulted in greater improvements in abdominal pain (83.2% vs. 69.6%), diarrhea (84.8% vs. 68.2%), and quality of life (84.1% vs. 74.5%) compared with standard treatment alone. The benefits were observed across all IBS subtypes, with the most pronounced reduction in abdominal pain reported in the IBS-D subgroup and significant quality-of-life improvements evident after 3 months of intervention [100]. The clinical relevance of this study lies in its pediatric focus, where safety profile and tolerability are of paramount concern in children, making postbiotic-containing formulations potentially effective not only in adults, but also in children.

### 5.3. Inflammatory Bowel Disease

Inflammatory bowel disease (IBD) includes Crohn’s disease and ulcerative colitis, both characterized by immune dysregulation and disruption of the epithelial barrier. Clinical and translational evidence suggests that postbiotics can reduce inflammatory activity by modulating cytokine activity and reinforcing the barrier. For example, tyndallized *Lactobacillus rhamnosus* significantly decreased inflammatory biomarkers in pediatric atopic conditions, indicating its potential for treating chronic inflammatory disorders like IBD [59]. Postbiotic administration decreased NF-κB signaling and increased Nrf2-mediated antioxidant pathways in inflammatory models, suggesting dual anti-inflammatory and cytoprotective effects [56]. These mechanisms align with therapeutic targets in managing IBD, focusing on suppressing pro-inflammatory cytokines (TNF-α, IL-6) and enhancing IL-10 production [101]. While large-scale RCTs in IBD are limited, current data show promising adjunctive potential.

Therapeutic mechanisms that consistently emerge across disease categories include reinforcement of intestinal barrier integrity, suppressing NF-κB-mediated inflammation, activation of Nrf2 antioxidant signaling, regulating SCFA metabolism, modulating bile acid and endocrine signaling, and restoring immune balance [102]. Guo et al. [103] reported strong intestinal health benefits of Pa-JY062, a novel postbiotic derived from probiotic-fermented milk. It improved immune response, reduced inflammation, strengthened gut barrier integrity, and enhanced microbiota diversity and SCFA production. Certain postbiotics have been shown to enhance gut health by reinforcing the intestinal barrier, reducing inflammation, and exerting antimicrobial effects against harmful gut microbes [91]. To enhance stability, bioavailability, and targeted intestinal delivery, postbiotics can be incorporated into advanced formulations such as hydrogel matrices or microencapsulation systems, which protect sensitive metabolites from gastrointestinal degradation and enable controlled release at specific gut sites. Importantly, postbiotics have a favorable safety profile and a lower risk of microbial translocation compared to live probiotics, making them suitable for immunocompromised or metabolically fragile populations [104]. A detailed summary of clinical and translational evidence is provided in Table 2.

### 5.4. Obesity and Metabolic Syndrome

Obesity is characterized by chronic low-grade inflammation, insulin resistance, altered gut permeability, and dysregulated lipid metabolism [121,122]. An RCT demonstrated that *Akkermansia muciniphila* postbiotic-fortified yogurt significantly reduced: waist circumference, body fat percentage, and serum liver enzymes [108]. The metabolites of postbiotics, such as bacteriocins, lipoteichoic acid, muramyl dipeptide, and lysates, perform anti-obesity activities by increasing energy expenditure, reducing adipogenesis and adipocyte differentiation, suppressing food intake, and regulating gut dysbiosis [123]. These findings indicate improvements in both adiposity and hepatic metabolic stress. Moreover, a four-week study in an obese pig model showed that supplementation with heat-killed *Ligilactobacillus salivarius* strain 189 (HK LS 189) demonstrated anti-obesity and gut microbiota-modulator properties. The intervention also reported lipid metabolism, excretory system function, metabolic pathways, and signal transduction pathways were significantly enhanced [124]. Wu et al. [125] reported that *Bacteroides*-derived postbiotics reduced hepatic lipogenesis gene expression and improved lipid and glucose parameters in obesity models. Mechanistically, these effects were linked to modulation of fatty acid oxidation pathways and suppression of inflammatory mediators. Additionally, SCFA-mediated activation of GPR41/43 enhances satiety signaling via GLP-1 secretion [25,26], contributing to appetite regulation and improved metabolic homeostasis. Yoon et al. [109] showed that S-layer proteins (SLPs) along with heat-killed *Lactobacillus curvatus* and *L. plantarum* inhibited adipocyte differentiation by reducing lipid accumulation and decreasing the expression of adipogenic markers such as peroxisome proliferator-activated receptor gamma (PPARγ) and fatty acid-binding protein 4 (FABP4), and induced apoptosis by increasing the activity of caspase-3. Similarly, Zhang et al. [111] reported that exopolysaccharides (EPS) produced by *Lactobacillus rhamnosus* GG inhibited the expression of several important genes associated with lipogenesis and adipogenesis in 3T3-L1 adipocytes, such as PPARγ, fatty acid synthase, lipoprotein lipase, and stearoyl-CoA desaturase-1, and decreased the accumulation of triglycerides in 3T3-L1 adipocytes, as well as the mass of adipose tissue and serum triglycerides and hepatic lipid deposition in high-fat diet-fed mice. Lee et al. [112] showed that EPS produced by *L. plantarum* L-14 suppressed the differentiation of adipocytes and the accumulation of lipids in these cells via activation of the AMP-activated protein kinase (AMPK) signaling pathway and the repression of adipogenic transcription factors, including CCAAT/enhancer-binding protein alpha (C/EBPα) and PPARγ. Collectively, clinical and mechanistic data suggest that postbiotics target multiple pathological axes of obesity: adipose tissue inflammation, hepatic lipid accumulation, insulin resistance, and gut barrier dysfunction.

### 5.5. Type 2 Diabetes and Prediabetes

Insulin resistance and impaired glucose metabolism are central to type 2 diabetes mellitus. Beteri et al. reported that postbiotic supplementation in prediabetic individuals significantly reduced HbA1c and fasting plasma glucose levels [78]. These improvements were associated with an increased abundance of butyrate-producing bacteria and enhanced SCFA levels. SCFAs improve glycemic control through multiple pathways including activation of AMPK, inhibition of hepatic gluconeogenesis, enhancement of insulin sensitivity, and epigenetic modulation via histone deacetylase inhibition [87]. Furthermore, Mori et al. demonstrated that postbiotic modulation of bile acid receptors, such as FXR and TGR5, improves glucose and lipid metabolism [27]. Together, these findings indicate that postbiotics influence both pancreatic insulin dynamics and hepatic metabolic regulation, suggesting potential as an adjunct in diabetes management. Additionally, heat-killed *L. plantarum* OLL2712 showed a beneficial effect on blood glucose regulation through modulation of inflammatory signaling [126]. A recent study with high-fat diet-fed mice showed that heat-treated *L. plantarum* HK L-137 was able to decrease adipose tissue inflammation, weight gain, and liver protection, which was partially attributed to its ability to strengthen the intestinal barrier and inhibit endotoxin translocation into the bloodstream [127].

### 5.6. Neurological Disorders

Postbiotics are also being identified as potential bioactive agents that modulate the gut–brain axis, the bidirectional communication system between the intestinal microenvironment and the central nervous system, which relies on neural, endocrine, and immune pathways. Although significant clinical evidence exists for their use in the context of stress, anxiety, and sleep-related outcomes in healthy or occupationally stressed adults, evidence is more limited in diagnosed neurological disease [128,129]. Heat-inactivated *Lactobacillus gasseri* CP2305 improved sleep quality and blunted exam-related cortisol elevation in medical students over 12 weeks [128]. Furthermore, a related crossover study reported that a single dose of heat-treated *Lactobacillus gasseri* CP2305 altered EEG alpha oscillatory activity, providing direct EEG evidence of a postbiotic-brain link in addition to symptom scales [129]. Similarily, a four-week administration of heat-killed *Lactiplantibacillus plantarum* SNK12 enhanced subjective sleep indices and reduced the salivary cortisol and plasma TNF-α levels [130], suggesting that modulation of the HPA axis and inflammatory pathways is not an artifact of a single strain but can also take place across different nonviable strains.

These findings collectively place postbiotics in a compelling position as possible safe agents, with potential utility in gut–brain-axis modulation, although the evidence base is still limited in terms of scope and population. Postbiotic and probiotic strains can be used in combination to achieve beneficial effects in a single gut–brain axis intervention. An 8-week trial of combination therapy with live *Lactiplantibacillus plantarum* PS128 and heat-treated *Lacticaseibacillus paracasei* PS23 resulted in decreases in job-stress perception, state anxiety, and insomnia severity, as well as reductions in serum ACTH and norepinephrine [77], demonstrating that this strain combination may be effective. One of the few clinical trials designed to isolate the relative contribution of the microbial viability from the intrinsic bioactivity of postbiotics employed a direct head-to-head design comparing a live dual-strain probiotic formulation with its nonviable, heat-inactivated postbiotic counterpart in adults reporting anxiety symptoms, assessing mood, vitality, and perceived stress outcomes. Overall, the available evidence is biologically plausible for postbiotics as a new dimension of gut–immune–metabolic axis therapeutics that can be explored in a controlled fashion in patients suffering from specific neurological or major psychiatric disorders. This is recognized as a key gap in the field and is highlighted here as a priority area for future controlled studies.

### 5.7. Liver Disease

Chronic liver diseases, including non-alcoholic fatty liver disease (NAFLD) and alcohol-related liver disease, are closely linked to gut dysbiosis and increased intestinal permeability. Hansen et al. demonstrated that ReFerm^®^ postbiotic supplementation significantly improved intestinal permeability in patients with alcohol-related liver disease by reducing endotoxin translocation, thereby decreasing hepatic inflammatory burden and slowing fibrosis progression [50]. Guerrero-Encinas et al. further showed that postbiotic IC-431 reduced mitochondrial oxidative stress in hepatic tissue, improving mitochondrial respiration efficiency [72]. Postbiotics may therefore benefit liver disease through reduction in endotoxemia, suppression of hepatic inflammation, restoration of mitochondrial function, and regulation of bile acid signaling pathways [62]. These mechanisms are highly relevant to NAFLD and metabolic-associated fatty liver disease (MAFLD). The clinical trials, including types of postbiotics, preparation methods, interventions, and intended benefits in reshaping immune, gut, and metabolic health, are summarized in Table 3.

## 6. Translational Challenges, Standardization, Regulatory Considerations, and Future Directions

Despite rapidly expanding mechanistic and clinical evidence supporting the therapeutic potential of postbiotics, significant translational barriers remain before their widespread clinical integration can be fully realized. The field is currently at an inflection point: while proof-of-concept studies demonstrate strong biological plausibility and encouraging therapeutic outcomes, several critical challenges must be addressed to facilitate the transition from experimental findings to clinically validated interventions.

### 6.1. Standardization of Postbiotic Preparations

One of the most pressing challenges in postbiotic research lies in the heterogeneity of formulations. Postbiotics encompass a broad spectrum of bioactive entities, including inactivated whole-cell preparations, cell wall fragments, exopolysaccharides, bacteriocins, extracellular vesicles, and metabolites such as SCFAs [17,18]. Differences in heat treatment conditions, fermentation substrates, bacterial strains, and downstream processing significantly influence the concentration of bioactive compounds, biological potency [21], and mechanistic actions against specific diseases [134,135]. Without standardized production parameters, reproducibility across studies remains limited. This also weakens causal interpretation, because observed biological effects cannot always be attributed to a specific microbial component, metabolite, or preparation method.

Clinical trials have demonstrated metabolic benefits, yet optimal dosing strategies, treatment durations, and long-term safety profiles are insufficiently characterized [84,108]. Harmonization of manufacturing protocols, compositional fingerprinting (e.g., metabolomics, proteomics), and bioactivity assays is urgently needed. The ISAPP has provided a working definition of postbiotics; however, regulatory and research communities must now move toward operational standards that define minimal characterization requirements for publication and commercialization [15]. At minimum, future studies should report microbial strain identity, viability testing after inactivation, preparation method, compositional profile, dose, stability data, and predefined biological activity markers.

### 6.2. Regulatory Classification and Safety Considerations

Regulatory ambiguity presents another major translational barrier. Depending on the jurisdiction, postbiotics may be classified as dietary supplements, functional foods, medical foods, and biotherapeutic agents. This classification significantly influences the evidentiary burden required for market approval. Unlike live probiotics, postbiotics do not carry risks of microbial translocation or opportunistic infection, particularly in immunocompromised individuals [13,14]. This safety advantage strengthens their case as next-generation microbiome therapeutics.

Nevertheless, regulatory agencies require clear demonstration of safety, compositional stability, and absence of residual viable organisms. Heat-killed preparations provide supportive safety data, but larger, multicenter studies are required to establish comprehensive pharmacovigilance profiles [51]. Safety evaluation should not be limited to microbial nonviability. It should also include residual endotoxin activity, immune stimulation, metabolite stability, contaminants, and unintended effects in vulnerable populations, such as children, older adults, pregnant women, and immunocompromised patients. Additionally, the mechanistic pleiotropy of postbiotics, impacting immune, metabolic, and endocrine pathways, raises important questions regarding long-term immunomodulatory effects. While modulation of NF-κB and Nrf2 signaling appears beneficial [20,56], prolonged immune manipulation warrants careful evaluation to avoid unintended immunological imbalance. Therefore, clinical translation requires not only short-term tolerability data but also long-term monitoring of immune, metabolic, hepatic, and gastrointestinal outcomes.

### 6.3. Mechanistic Complexity and Precision Medicine

Postbiotics have multilayered biological effects, engaging intestinal epithelial signaling (PI3K/Akt), immune receptors (TLRs, NOD), GPCR-mediated SCFA pathways (GPR41/43), bile acid receptors (FXR, TGR5), and epigenetic regulators such as histone deacetylases [22,66]. This mechanistic breadth presents both an opportunity and a challenge. Although these pathways provide plausible biological explanations, most studies demonstrate association rather than direct causality. Therefore, mechanistic claims should be interpreted according to strength of the experimental model and the specificity of the intervention.

While pleiotropy enhances therapeutic potential across metabolic and immune disorders, it complicates mechanistic attribution. For example: Barrier reinforcement [22], cytokine modulation [24,55], antioxidant activation via Nrf2 [56], and endocrine regulation, including GLP-1 and sex hormones [94,95]. In multi-component preparations, it is often unclear whether the observed effect is driven by inactivated cells, cell wall structures, extracellular vesicles, metabolites, or interaction among these components. These pathways may operate simultaneously, making it difficult to isolate primary mechanisms of action. Future research should integrate multiomics approaches (e.g., metagenomics, metabolomics, transcriptomics, and proteomics) to identify biomarker signatures predictive of therapeutic response. Precision microbiome stratification may enable targeted use of specific postbiotic formulations tailored to individual metabolic or immune phenotypes. This is particularly important because baseline microbiota composition, diet, age, disease severity, medication use, and host metabolic status may strongly influence postbiotic responsiveness.

### 6.4. Need for Large-Scale Randomized Controlled Trials

Although promising clinical outcomes have been reported in IBS [51], obesity [108], prediabetes [79], liver disease [50], and immune disorders [64], many studies remain limited by small sample sizes and short follow-up durations. Large, multicenter RCTs are necessary to confirm efficacy across diverse populations, determine long-term safety, define standardized dosage regimens, compare postbiotics directly with live probiotics, and evaluate additive or synergistic effects with pharmacotherapy. To date, no published clinical trial has directly evaluated whether combining postbiotics with prebiotics and/or probiotics provides greater therapeutic benefit than postbiotic monotherapy in the management of gut dysfunction. The closest available evidence is provided by Wanitsuwan et al. [130], who assessed postbiotic and live-probiotic interventions in separate parallel arms rather than as a combined treatment. Their findings demonstrated statistically comparable effects on inflammatory and metabolic biomarkers, with no evidence of a synergistic advantage. Consequently, in the absence of rigorously designed combination-versus-monotherapy trials, the proposed benefits of integrating postbiotics, prebiotics, and probiotics remain biologically plausible but clinically unsubstantiated.

Future RCTs should be designed to test for causality as well as clinical association. This demands adequate statistical power, harmonization of clinical and mechanistic outcome measures, formulation standardization, and the inclusion of predefined primary endpoints, as well as the use of a placebo or active comparator. In addition, future trials need to incorporate mechanistic endpoints, such as markers of gut permeability, the measurement of inflammatory cytokines, quantification of SCFAs, metabolomic profiling, and metabolic biomarkers, to bolster causal inference. Subgroup analyses should also be included to assess whether treatment effects vary according to age, sex, baseline microbiota composition, metabolic phenotype, disease stage, and dietary pattern. If this stratification is not performed, clinical significance could be weakened or be misinterpreted within a heterogeneous population. Although postbiotics are generally considered safe and may have potential as adjunctive interventions, clinical evidence regarding their simultaneous administration with disease-specific protective agents remains limited. Therefore, possible interactions, altered therapeutic responses, or unforeseen complications require systematic investigation in future clinical studies.

### 6.5. Integration into Metabolic and Immune Therapeutic Frameworks

The intersection of gut barrier integrity, immune modulation, oxidative stress regulation, and endocrine signaling positions postbiotics as integrative therapeutics in chronic disease management. In metabolic disorders, postbiotics influence insulin sensitivity, lipogenesis and fatty acid oxidation [88,97], bile acid signaling [27], and adiposity and hepatic enzymes [108]. In immune-mediated conditions, they modulate Th1/Th2 balance, Treg/Th17 equilibrium [67], NK cell activity [64], and dendritic cell activation [65,66]. This integrative capacity suggests that postbiotics may function not merely as adjuncts but as core components of microbiome-centered therapeutic strategies.

## 7. Future Directions

Future investigations should prioritize several key areas to fully realize the therapeutic potential of postbiotics. These areas include standardized manufacturing and compositional profiling, dose–response characterization, long-term safety surveillance, integration of multiomics biomarkers, development of precision-targeted postbiotic formulations, and regulatory harmonization across jurisdictions. However, these priorities should not be viewed only as technical improvement; they directly address the major translational barriers that currently limit interpretation of the evidence. Emerging delivery technologies, such as microencapsulation, polymeric hydrogels, and synthetic biology-derived carriers, may enhance stability, protect bioactive metabolites from gastrointestinal degradation, enable controlled release at specific intestinal sites, and improve therapeutic precision [104].

These strategies can facilitate colonization-independent activity and tailored dosing, expanding the clinical applicability of postbiotics in diverse populations. Additionally, exploring postbiotics in neuroimmune and gut–brain axis disorders represents a promising frontier, given established links between SCFAs, inflammatory signaling, and central nervous system function [53,54]. Mechanistically, postbiotics reinforce intestinal barrier integrity, modulate immune responses, restore redox balance, and regulate metabolic–endocrine pathways, collectively positioning them as next-generation microbiome-based therapeutics. Despite these advances, translation to clinical practice will require rigorous standardization, mechanistic validation, and large-scale RCTs. Such trials should use clearly characterized interventions, predefined mechanistic endpoints, harmonized outcome measures, adequate follow-up periods, and transparent reporting of formulation, dose and administration route. Without these methodological safeguards, positive findings may remain difficult to replicate and may not provide sufficient evidence for regulatory approval or clinical adoption. Coordinated scientific and regulatory efforts, alongside advanced delivery platforms, are therefore essential to unlock the full potential of postbiotics. With these integrated approaches, postbiotics are poised to become foundational components of precision interventions for chronic metabolic, inflammatory, and neuroimmune disorders.

## 8. Limitations of the Study

Beyond methodological limitations of individual studies, the broader field of postbiotic research faces several unresolved challenges. The bioavailability and pharmacokinetics of many postbiotic compounds remain poorly understood, and it is unclear to what extent these molecules reach systemic circulation after intestinal administration. This creates an important translational gap because biological activity observed in cell-based or animal models cannot automatically be assumed to occur in humans at clinically achievable concentrations. The inter-individual variability in gut microbiota composition may influence host responsiveness to postbiotic interventions. The lack of a dose–response relationship makes it difficult to draw causal inferences, as it is unclear whether any observed benefits are actually due to the biological properties of the drug, or due to a threshold effect or variability in the studies. The complex postbiotics are poorly understood during processing, storage, and digestion. This has a direct impact on the reproducibility of the product, as two preparations that are labeled as postbiotics may have significantly different molecular composition, bioactive metabolite concentrations, and physiological effects.

The stability of complex postbiotic preparations during processing, storage, and gastrointestinal transit remains insufficiently characterized. Addressing these issues will be essential for translating mechanistic findings into clinically reproducible interventions. Despite the growing body of evidence supporting the therapeutic potential of postbiotics, several limitations should be acknowledged in the current review. The available literature remains highly heterogeneous with respect to postbiotic composition, microbial strains, inactivation methods, and dosage regimens, making direct comparisons across studies challenging. A substantial proportion of mechanistic evidence is derived from in vitro experiments and animal models, which may not fully reflect physiological responses in humans. Although an increasing number of clinical studies have been reported, many trials involve relatively small sample sizes and short intervention periods, limiting the ability to draw firm conclusions regarding long-term efficacy and safety. In addition, variability in study design, outcome measures, and reporting standards further complicates the synthesis of clinical evidence. The lack of standardized definitions, production protocols, and regulatory frameworks for postbiotic preparations remains a major barrier to translating experimental findings into consistent clinical applications. Future studies should not only report strain identity and inactivation procedures but also provide detailed molecular characterization, batch-to-batch quality control, dose–response analysis, and validated biomarkers of biological activity. Future research should therefore prioritize well-designed, large-scale randomized controlled trials (RCTs), standardized characterization of postbiotic formulations, and harmonized reporting guidelines to strengthen the evidence base and facilitate clinical translation.

## 9. Conclusions

Postbiotics represent a promising advancement in microbiome-based therapeutics, providing stable and safe bioactive preparations that may influence the gut–immune–metabolic axis. Research suggests that postbiotics may improve intestinal barrier integrity, modulate inflammatory responses, support antioxidant pathways, and contribute to metabolic regulation through various mechanisms. Emerging clinical studies have reported potential benefits of postbiotics in conditions such as IBS, obesity, prediabetes, liver disease, and immune disorders, with observed improvements in selected metabolic, inflammatory, gut health, and immune-related outcomes. Importantly, postbiotics may offer safety advantages compared with some live microbial treatments, particularly for vulnerable populations. However, further clinical evaluation is required to establish their comparative safety profiles. Further progress in research and development will require standardized production methods, detailed characterization, large-scale RCTs, and regulatory alignment. By integrating advanced technologies and personalized microbiome profiling, postbiotics may be further optimized for more effective interventions. Moreover, postbiotics have shown potential to modulate complex host signaling networks related to intestinal health, immune function, oxidative stress, and metabolism. With ongoing scientific advancements and collaborative research, postbiotics may become valuable components of integrative strategies for managing chronic metabolic and immune conditions. Moreover, ABB C1^®^ is a formulation composed of yeast-derived ingredients, including a distinctive β-1,3/1,6-glucan complex and a postbiotic consortium of *Saccharomyces cerevisiae* enriched with selenium and zinc. This supplement has been reported to exhibit anti-inflammatory effects, support intestinal barrier function, and stimulate phagocytic activity [136]. A previous RCT suggested that ABB C1^®^ may provide potential clinical benefits as a dietary supplement during immune challenges such as infections or allergic reactions, with possible effects on symptom reduction and gastrointestinal outcomes [137]. However, further independent clinical studies are needed to confirm its efficacy and define its therapeutic applications.

## Figures and Tables

**Table 1:** Postbiotics, targeted effects, mechanistic pathways, evidence strength in treating metabolic disorders.

Therapeutic Targets	Postbiotic Composition	Study Type	Mechanistic Pathway	Evidence Strength	Reference
Antihyperglycemic activity	Fermentation-derived metabolites of *Lactiplantibacillus plantarum* R6-3	In vitro/Animal	Stimulation of GLP-1, improvement of fasting insulin and modulation of the gut microbiome–GLP–1–muscle/adipose/liver axis, and inhibition of α-glucosidase and α-amylase.	Moderate	[73]
Lower systemic inflammation	Extracellular vesicles derived from Escherichia coli A0 34/86 (EcO83)	Animal	Downregulation of TNF-α and IL-6, and prevention of nosocomial infections and diarrhea in newborns, stimulation of both pro- and anti-inflammatory cytokine production, and its intranasal administration reduces allergic airway inflammation in mice.	Low-moderate	[74]
Improved muscle strength and gut microbiota modulation	Whey peptides, kefir LAB-derived SCFAs, polyphenol metabolites, and kefir LAB-derived extracellular vesicles (*Lentilactobacillus kefiri* DH50)	Animal	Enhancement of the gut–muscle axis via increased irisin levels and reduced IL-1β; modulation of microbiota composition by enriching beneficial taxa (e.g., *Faecalibacterium prausnitzii*), contributing to anti-inflammatory effects	Low	[75]
Reduced oxidative stress and inflammation	Short-chain fatty acids and exopolysaccharides from *Lactobacillus paracasei*	Animal	Reduction in IL-1β, MDA, and hs-CRP, along with elevation of TAC; enhancement of antioxidant defense and reduction in infection risk (pneumonia)	Moderate	[76]
Improved gut muscle strength and reduced inflammation	Microcrystalline cellulose, live *L. paracasei* PS23 group, and heat-treated *Lacticaseibacillus paracasei* PS23 (HT-PS23)	Animal	Reduction in inflammatory markers (CRP, IL-6), elevation of IL-10 and testosterone levels, and enhancement of muscle function via anti-inflammatory and endocrine modulation	Low	[77]
Improved glucose metabolism in prediabetes	Konjac glucomannan, galacto-oligosaccharides, and exopolysaccharides from *Bifidobacterium breve*	Human	Enhancement of insulin sensitivity and glucose metabolism through increased abundance of butyrate-producing bacteria and improved gut barrier integrity	High	[78]
Enhanced amino acid absorption	γ-irradiated (postbiotic) preparations of *Lacticaseibacillus paracasei*	Animal	Improvement of intestinal absorption kinetics of amino acids via modulation of gut metabolic activity and nutrient transport mechanisms	Low	[79]
Improved gut microbiome architecture and metabolic function	KLP-KM2 heat-treated *Lactiplantibacillus plantarum* KM2 fermentation-derived postbiotic	Animal	Promotion of microbial diversity and colonization of beneficial taxa (e.g., *Veillonella* spp.), supporting energy metabolism and host–microbiome interactions	Low-moderate	[80]
Improved gut health and lipid metabolism	Heat-treated postbiotic of *Bifidobacterium* CECT 7347 (HT-ES1) capsules	Human	Increase in butyrate-producing bacteria (*Faecalibacterium, Anaerobutyricum*), stabilization of gut inflammation (calprotectin), and reduction in serum cholesterol levels	High	[81]
Improved lipid metabolism and gut microbiota in NAFLD	Postbiotics prepared from *Lactobacillus paracasei* CCFM1224	Animal	Improvement of gut microbiota composition and regulation of hepatic metabolic pathways, including glycerophospholipid metabolism, lipid metabolism, pantothenate and CoA biosynthesis, and amino acid metabolism, mediated through modulation of gene expression in hepatocytes at the mRNA level	Low-moderate	[82]

Evidence strength was classified according to the level of available scientific evidence: high strength indicates findings supported by human clinical studies; moderate strength indicates evidence from animal studies with consistent mechanistic outcomes and/or limited clinical validation; low-to-moderate strength represents evidence mainly derived from animal studies with partial mechanistic support; and low strength indicates evidence primarily based on preclinical models (animal or in vitro studies) without human clinical confirmation. Abbreviations used: CRP, C-reactive protein; EcO83, *Escherichia coli* A0 34/86; GLP-1, glucagon-like peptide-1; hs-CRP, high-sensitivity C-reactive protein; HT-PS23, heat-treated *Lacticaseibacillus paracasei* PS23; IL-1β, interleukin-1 beta; IL-6, interleukin-6; IL-10, interleukin-10; KM2, *Lactiplantibacillus plantarum* KM2; MDA, malondialdehyde; NAFLD, nonalcoholic fatty liver disease; SCFAs, short-chain fatty acids; TAC, total antioxidant capacity; TNF-α, tumor necrosis factor-α.

**Table 2:** Summary of clinical, preclinical, and translational studies evaluating postbiotics in metabolic and immune disorders.

Disease Condition	Feature/Strategy	Postbiotic	Study Design	Example Approach	Observed Effects	Reference
Irritable Bowel Syndrome	*Bifidobacterium longum* CECT 7347	Heat-treated bacteria (paraprobiotic/whole-cell postbiotic)	Randomized controlled trial	2 capsules (ES1, HT-ES1 or placebo) daily for 12 weeks	Improvement in IBS-SSS score, stool consistency, quality of life, abdominal pain, and anxiety	[51]
Constipation	NextDext^TM^/ABB C22	Microbial-derived functional preparation (prebiotic–postbiotic mixture)	Double-blind RCT	2-week supplementation in 24 participants	Increase in beneficial bacteria (*B. longum*, *Roseburia hominis*); decrease in constipation-associated taxa (*Blautia obeum*, *E. coli*); improvement of gut microbial balance	[105]
Diarrhea	Probio-Eco	Cell-free postbiotic/microbial metabolite preparation	Crossover placebo-controlled trial	21-day intervention with washout	Improvement in stool form, defecation frequency, urgency, anxiety; increase in beneficial microbiota; reduction in pathogenic microorganisms	[106]
Ulcerative colitis	SCFAs, tryptophan metabolites	Microbial metabolites (postbiotic bioactive compounds)	Mechanistic/translational study	Experimental pathway analysis	Modulation of immune response through gut–immune signaling pathways	[107]
Diabetes (prediabetes)	KGM, GOS and EPSs from *Bifidobacterium breve*	Postbiotic and prebiotic mixture	Placebo-controlled RCT	12-week intervention (*n* = 53)	Increase in alpha diversity and butyrate-producing bacteria; reduction in HbA1c and fasting plasma glucose levels	[79]
Obesity/Metabolic dysfunction	*Akkermansia muciniphila*, *Lactobacillus rhamnosus*	Heat-killed bacteria/postbiotic-fortified food matrix	Randomized double-blind trial	8-week supplementation (*n* = 66)	Reduction in anthropometric and metabolic markers including waist circumference, fat mass, AST levels, and appetite scores	[108]
Stress-induced ovarian dysfunction	GABA + *Lactobacillus plantarum* 1-2-3	Microbial metabolite (GABA) and bacterial intervention	Animal experimental model	Stress-induced ovarian dysfunction model	Reduction in oxidative stress and inflammation; elevation of reproductive hormones; modulation of steroidogenesis-related pathways	[95]
Obesity/Adipogenesis	*Lactobacillus curvatus* and *Lactobacillus plantarum*	S-layer proteins and heat-killed bacteria	In vitro cell model	3T3-L1 adipocytes (48 h treatment)	Reduction in lipid accumulation, downregulation of adipogenic markers (FABP4, PPARγ), decreased BCL-2, enhancement of caspase-3-mediated apoptosis, and inhibition of adipocyte maturation	[109]
Inflammation	*Bifidobacterium longum* CECT-7347	Heat-treated bacteria (paraprobiotic)	In vitro cell study	HT-29 intestinal epithelial cells	Reduction in oxidative stress and inflammation; improved gut barrier integrity; prevention of pathogen colonization and preservation of antioxidant activity	[110]
Obesity and diet-induced metabolic dysfunction	*Lactobacillus rhamnosus* GG	Exopolysaccharides	In vitro + animal study	3T3-L1 cells + HFD mice	Reduction in intracellular TAG accumulation, adipocyte differentiation, fat mass, serum and hepatic lipid levels; downregulation of adipogenic and lipogenic genes including PPARγ, aP2, FAS, SCD1, LPL, and DGAT1	[111]
Liver fibrosis	ReFerm^®^	Inactivated microbial cell matrix and metabolites (fermented postbiotic)	Randomized controlled trial	24-week supplementation (*n* = 56)	Reduction in liver fibrosis progression through enhancement of gut barrier function	[50]
Supporting gut health	*Lactiplantibacillus plantarum* KM2 (KLP-KM2)	Heat-treated bacteria/fermented postbiotic matrix	Double-blind RCT	12-week elderly intervention	Promotion of de novo colonization of *Veillonella* spp.	[81]
Obesity/Metabolic syndrome	*Lactobacillus plantarum* L-14	Exopolysaccharides	In vitro study	3T3-L1 and MSC cell models	Reduced TAG storage and adipocyte differentiation; suppressed PPARγ, C/EBPα, FAS, LPL, CD36, GPDH, and SREBP-1c expression; enhanced AMPK and AKT signaling and increased adiponectin levels	[112]
Halitosis	*Lactobacillus paracasei* ET-22 (live + heat-killed)	Heat-killed bacteria (paraprobiotic)	Randomized placebo-controlled trial	Lozenges for 4 weeks	Reduction in oral pathogens (*Rothia*, *Streptococcus*, *Solobacterium*) and oral malodor	[113]
Obesity	*Bifidobacterium breve* BB091109	Postbiotic extract (cell-derived bioactive fraction)	Randomized double-blind trial	12-week supplementation	Improvement of endocrine function and anti-inflammatory status	[94]
Obesity/Adiposity	*Bifidobacterium animalis* subsp. *lactis* BPL1	Cell wall component (lipoteichoic acid)	*C. elegans* obesity model	Purified LTA administration	Reduction in fat deposition through modulation of the insulin/IGF-1 signaling pathway	[114]
Cardiovascular Dysfunction Associated with Aging	BPL1^TM^-HT (heat-inactivated *Bifidobacterium animalis* subsp. *lactis* BPL1)	Heat-inactivated bacteria/Whole-cell postbiotic	Animal study (aged rats)	Dietary supplementation	Attenuation of age-associated cardiovascular alterations, reduction in oxidative stress and inflammation, and improvement of vascular function	[115]
Osteoarthritis-Associated Inflammation	Heat-treated *Lacticaseibacillus rhamnosus* strains	Heat-treated bacteria/Whole-cell postbiotic	In vitro screening	Cell-based inflammatory assays	Modulation of inflammatory and metabolic pathways associated with joint health and osteoarthritis progression	[116]
Metabolic Syndrome	BPL1^®^-HT combined with complex tea extract	Heat-inactivated bacteria/Whole-cell postbiotic	Animal study	Combined dietary intervention	Improvement of cardiometabolic parameters, reduction in adiposity, enhancement of metabolic regulation, and attenuation of inflammatory markers	[117]
Type 2 Diabetes Mellitus	*Lactobacillus rhamnosus* DV-NRRL B-68023 lysate	Bacterial lysate (cell wall and DNA-derived postbiotic/metabiotic)	Randomized double-blind RCT	3-month oral supplementation (*n* = 55)	Significant reduction in HbA1c (−0.63%), improvement in glycemic control, reduction in waist circumference, and enhancement of insulin sensitivity compared with placebo	[118]
Obesity and Gut Metabolic Health	Heat-treated yogurt	Fermented food-derived postbiotic matrix (heat-treated fermented dairy)	Randomized controlled trial	16-week dietary intervention (*n* = 100)	Altered fecal metabolome characterized by reduced amino acids (leucine, valine, threonine) and isobutyrate, indicating modulation of gut microbial metabolism; effects comparable to conventional yogurt consumption	[119]
Abdominal Obesity/Cardiometabolic Risk	SIAP2	Heat-inactivated bacteria (whole-cell postbiotic)	Randomized double-blind RCT	12-week enriched food intake (*n* = 120)	Reduction in fasting insulin and HOMA-IR, improvement in insulin resistance, reduction in pulse pressure among women, attenuation of postprandial triglyceride response, and modulation of gut microbiota composition	[120]

Note: This is a summary of clinical trials, preclinical trials and translational studies that have evaluated the effects of postbiotic interventions on metabolic and immune-related outcomes. These outcomes include glycemic control, lipid metabolism, intestinal permeability, inflammatory biomarkers, and immune modulation. Abbreviations used: ABB C22, Advanced Biotic Blend C22; AKT, Protein Kinase B; AMPK, Adenosine Monophosphate-Activated Protein Kinase; aP2, Adipocyte Protein 2 (Fatty-Acid-Binding Protein 4); AST, Aspartate Aminotransferase; BCL-2, B-Cell Lymphoma 2; BPL1, *Bifidobacterium animalis* subsp. lactis CECT 8145; BPL1^®^-HT/BPL1^TM^-HT, Heat-Treated *Bifidobacterium animalis* subsp. lactis BPL1; C/EBPα, CCAAT/Enhancer-Binding Protein α; CECT, Colección Española de Cultivos Tipo (Spanish Type Culture Collection); DGAT1, Diacylglycerol O-Acyltransferase 1; DNA, Deoxyribonucleic Acid; DV-NRRL, Division of Veterinary–Northern Regional Research Laboratory; EPS, Exopolysaccharide; EPSs, Exopolysaccharides; ES1, Experimental Supplement 1; FABP4, Fatty Acid-Binding Protein 4; FAS, Fatty Acid Synthase; GABA, Gamma-Aminobutyric Acid; GOS, Galacto-Oligosaccharides; GPDH, Glycerol-3-Phosphate Dehydrogenase; HbA1c, Glycated Hemoglobin A1c; HFD, High-Fat Diet; HOMA-IR, Homeostatic Model Assessment of Insulin Resistance; HT, Heat-Treated; HT-29, Human Colon Adenocarcinoma Cell Line 29; HT-ES1, Heat-Treated Experimental Supplement 1; IBS, Irritable Bowel Syndrome; IBS-SSS, Irritable Bowel Syndrome Severity Scoring System; IGF-1, Insulin-Like Growth Factor 1; KGM, Konjac Glucomannan; KLP-KM2, *Lactiplantibacillus plantarum* KM2; LPL, Lipoprotein Lipase; PPARγ, Peroxisome Proliferator-Activated Receptor Gamma; ReFerm^®^, Fermented Oat and Barley-Based Postbiotic Product; SCD1, Stearoyl-CoA Desaturase 1; SCFAs, Short-Chain Fatty Acids; SIAP2, Seafood Stick Formulation Enriched with Heat-Inactivated *Bifidobacterium animalis* subsp. lactis CECT 8145, Inulin, and Omega-3 Fatty Acids; SREBP-1c, Sterol Regulatory Element-Binding Protein 1c; TAG, Triacylglycerol; *Veillonella* spp., *Veillonella* species.

**Table 3:** Clinical trials reporting postbiotics as reshaping immune, gut, and metabolic health.

Bacterial Strain/ Preparation	Type of Evidence	Type of Study	Study Population	Interventions/Model	Duration	Observed Outcomes	Reference
*Lactiplantibacillus plantarum*	Heat-treated bacteria	Randomized, double-blind, placebo-controlled trial	Healthy adults	Oral supplementation of capsules containing 5 × 10^11^ cells of HT-nF1	8 weeks	Improvement of natural killer cell activity, enhancement of innate immune function by regulation of Th1-related immune factors	[64]
*Lacticaseibacillus paracasei* MCC1849	Heat-killed bacteria	Randomized, double-blind, placebo-controlled trial	Healthy adults	Oral intake of powder containing 5 × 10^10^ heat-killed MCC1849 cells	4 weeks	Enhancement of host immune responses and improvement in the expression level of CD86 on pDCs and gene expression levels of IFN-α, -β, and -γ upon TLR9 agonist stimulation	[65]
*Lactobacillus helveticus* GCL1815	Heat-treated bacteria	Randomized, double-blind, placebo-controlled trial	Healthy adults	Capsules containing either six billion *L. helveticus* GCL1815 cells	8 weeks	Enhancement of CD86 expression on plasmacytoid dendritic cells, improvement in HLA-DR expression, and upregulation of antiviral responses through activation of two types of dendritic cells	[66]
*Bifidobacterium breve* bioactive compounds C50 and *Streptococcus thermophilus* ST065	Fermented infant formula (probiotic-fermented preparation/postbiotic-like matrix)	Randomized, double-blind, controlled, multi-center study	Infants	Fermented infant formula containing bioactive compounds and prebiotics such as scGOS/lcFOS (9:1)	4 months (with stool sampling continuing to 6 months)	Improvement in gut microbiota composition, enhancement of gut metabolic activity similar to that of breastfed child, and improvement of sIgA levels	[131]
*B. longum* CECT 7347	Heat-treated bacteria	Randomized, double-blind, placebo-controlled pilot trial	Healthy adults	Oral supplementation of two capsules of postbiotic HT-ES1 daily	8 weeks, with a further follow-up assessment at week 10	Stabilization of inflammatory markers, reduction in total and non-HDL cholesterol, increase in butyrate-producing bacteria in the gut, maintenance of normal levels of fecal calprotectin and reducing serum cholesterol levels.	[81]
*Lactobacillus helveticus* CP790	Heat-treated bacteria	Randomized, double-blind, placebo-controlled trial	Healthy adults	CP790-fermented milk, while the placebo beverages were prepared with non-fermented milk flavored with lactic acid.	4 weeks	Significant improvement in stool consistency and reduced straining during defecation. *Desulfobacterota* levels significantly decreased, with a decline in the proportion of genes involved in the sulfate reduction pathway	[132]
*Lacticaseibacillus paracasei* PS23	Heat-treated bacteria	Randomized, double-blind clinical trial	Elderly	Placebo (0 CFU/d), L-PS23 (live PS23, 2 × 10^10^ CFU/d), or HT-PS23 (heat-treated PS23, 2 × 10^10^ cells/d)	12 weeks	Reduction in inflammatory markers related to aging (CRP), interleukin-6, and modulation of interleukin-10, with enhancement of lower limb muscle strength and endurance	[77]
-	Fermentation-derived postbiotics	Pilot randomized controlled trial	Healthy ambulatory adult patients	Oral supplementation	10 days	Increase in microbial diversity, enhancement of beneficial taxa, and reduction in gastrointestinal infections induced by antibiotics	[133]

Abbreviations used: CFU, colony-forming units; CD86, cluster of differentiation 86; HLA-DR, Human Leukocyte Antigen—DR isotype; HT, heat-treated; HT-ES1, heat-treated *B. longum* CECT 7347; HT-nF1, heat-treated *Lactiplantibacillus plantarum* nF1; HT-PS23, heat-treated *Lacticaseibacillus paracasei* PS23; IFN, interferon; IL, interleukin; lcFOS, long-chain fructo-oligosaccharides; L-PS23, live *Lacticaseibacillus paracasei* PS23; pDCs, plasmacytoid dendritic cells; scGOS, short-chain galacto-oligosaccharides; sIgA, secretory immunoglobulin A; Th, T helper cells; TLR9, toll-like receptor 9.

## Data Availability

No new data were created or analyzed in this study. Data sharing is not applicable to this article.

## Abbreviations

The following abbreviations are used in this manuscript:

ABB C1	Advanced Biotic Blend C1
AD	Atopic Dermatitis
Akt	Protein Kinase B
AMPK	AMP-activated protein kinase
CAT	Catalase
CD86	Cluster of differentiation 86
CFU	Colony-forming units
CRP	C-reactive protein
DHEA	Dehydroepiandrosterone
EPS	Exopolysaccharides
FFA2	Free fatty acid receptor 2
FFA3	Free fatty acid receptor 3
FXR	Farnesoid X receptor
GABA	Gamma-aminobutyric acid
GALT	Gut-associated lymphoid tissue
GLP-1	Glucagon-like peptide-1
GOS	Galacto-oligosaccharides
GPCR	G-protein-coupled receptor
GPR41	G-protein-coupled receptor 41
GPR43	G-protein-coupled receptor 43
GPR109A	G-protein-coupled receptor 109A
GPx	Glutathione peroxidase
HbA1c	Glycated hemoglobin
HDAC	Histone deacetylase
HKB	Heat-killed bacteria
HLA-DR	Human leukocyte antigen-DR
IBD	Inflammatory bowel disease
IBS	Irritable bowel syndrome
IBS-D	Diarrhea-predominant irritable bowel syndrome
IBS-SSS	Irritable bowel syndrome symptom severity score
IFN	Interferon
IFN-γ	Interferon gamma
IκB	Inhibitor of nuclear factor kappa B
IL-1β	Interleukin-1 beta
IL-6	Interleukin-6
IL-10	Interleukin-10
IL-12	Interleukin-12
ISAPP	International Scientific Association for Probiotics and Prebiotics
KGM	Konjac Glucomannan
LPS	Lipopolysaccharide
MAPK	Mitogen-activated protein kinase
MAFLD	Metabolic-associated fatty liver disease
MDA	Malondialdehyde
MeSH	Medical subject headings
MUC2	Mucin-2
NAFLD	Non-alcoholic fatty liver disease
NF-κB	Nuclear factor kappa B
NK	Natural killer cell(s)
NOD	Nucleotide-binding oligomerization domain
Nrf2	Nuclear factor erythroid 2-related factor 2
PI3K	Phosphoinositide 3-kinase
PRR	Pattern recognition receptor
PYY	Peptide YY
RCT(s)	Randomized controlled trial(s)
ROS	Reactive oxygen species
SCFA(s)	Short-chain fatty acid(s)
scGOS	Short-chain galacto-oligosaccharides
SOD	Superoxide dismutase
TGR5	G-protein-coupled bile acid receptor 1
Th	T helper cells
TJ	Tight junction
TLR	Toll-like receptor
TNF-α	Tumor necrosis factor alpha
